# Coronavirus Disease Case Definitions, Diagnostic Testing Criteria, and Surveillance in 25 Countries with Highest Reported Case Counts

**DOI:** 10.3201/eid2801.211082

**Published:** 2022-01

**Authors:** Amitabh B. Suthar, Sara Schubert, Julie Garon, Alexia Couture, Amy M. Brown, Sana Charania

**Affiliations:** Centers for Disease Control and Prevention, Atlanta, Georgia, USA

**Keywords:** COVID-19, coronavirus disease, SARS-CoV-2, severe acute respiratory syndrome coronavirus 2, viruses, respiratory infections, zoonoses

## Abstract

We compared case definitions for suspected, probable, and confirmed coronavirus disease (COVID-19), as well as diagnostic testing criteria, used in the 25 countries with the highest reported case counts as of October 1, 2020. Of the identified countries, 56% followed World Health Organization (WHO) recommendations for using a combination of clinical and epidemiologic criteria as part of the suspected case definition. A total of 75% of identified countries followed WHO recommendations on using clinical, epidemiologic, and diagnostic criteria for probable cases; 72% followed WHO recommendations to use PCR testing to confirm COVID-19. Finally, 64% of countries used testing eligibility criteria at least as permissive as WHO. We observed marked heterogeneity in testing eligibility requirements and in how countries define a COVID-19 case. This heterogeneity affects the ability to compare case counts, transmission, and vaccine effectiveness, as well as estimates derived from case surveillance data across countries.

Novel infectious pathogens can pose major challenges to global health and security. Tracking the geography, demographics, and suspected mode of transmission of these pathogens by using a standardized case definition remains the foundation for infectious disease surveillance (*1*). Severe acute respiratory syndrome coronavirus 2 (SARS-CoV-2), the virus that causes coronavirus disease (COVID-19), was first characterized in December 2019 (*2*). By January 2020, the first national case definition was developed (*3*) and the World Health Organization (WHO) declared a public health emergency of international concern (*4*). WHO’s interim guidance for global COVID-19 surveillance, released on January 31, 2020, provided a hierarchy of confirmed, probable, and suspected case definitions (*5*). This guidance encouraged the use of all available clinical, epidemiologic, and laboratory evidence for case classification purposes and noted that countries might need to adapt these definitions to their unique epidemiologic situations. Recommendations for testing suspected cases and widespread testing on the basis of transmission intensity, number of cases, and available resources were included.

Both WHO and national case definitions have evolved as knowledge about COVID-19 etiology and the myriad of ways the disease manifests after infection has grown (*5*–*7*). Early on, surveillance emphasized a travel history to Wuhan, China, where the initial outbreak occurred, and a narrowly defined set of symptoms. However, the virus rapidly spread to other provinces in China and then internationally, and reports of patients who experienced new symptoms or remained asymptomatic increased (*8*). Confirming a COVID-19 case relies on diagnostic testing; therefore, testing capacity has played a vital role in COVID-19 surveillance efforts. The types of tests available have expanded to include molecular and antigen tests to detect the presence of the SARS-CoV-2 virus and serologic tests to detect antibodies produced from previous SARS-CoV-2 infection (*9*,*10*). However, the availability of these tests and the resources needed to collect, handle, and process clinical specimens have varied widely across nations (*11*). Shortages of test kits and reagents and lack of laboratory capacity have forced officials in many locations to make difficult decisions about testing eligibility (*12*).

Differences in testing eligibility criteria and case definitions pose a challenge not only to detecting the actual number of cases within countries, but also to understanding the global burden of disease and adequately responding to pandemics. Global guidelines have been developed for testing eligibility criteria and case definitions but are usually reviewed at a national level and are subject to adaptation on the basis of laboratory and health system considerations. Earlier evaluations of global COVID-19 case definitions do not reflect the latest changes to national case definitions and testing eligibility criteria and do not target the full range of countries with the highest number of reported COVID-19 cases (*13*–*15*). We analyzed national COVID-19 case definitions from the 25 countries with the largest number of reported COVID-19 cases as of October 1, 2020 (collectively representing ≈85% of the global cases at that time), and the specific criteria used to determine eligibility for diagnostic testing. We also determined the implications of intercountry differences on ongoing efforts to understand global disease burden and control the pandemic.

## Methods

### Design

We identified the 25 countries with the highest number of reported COVID-19 cases from WHO COVID-19 cumulative case counts as of October 1, 2020 (*16*). We extracted surveillance case definitions and official testing policies from official government Web sites for the respective countries. If definitions were not available on government Web sites, we extracted definitions from personal communication with Centers for Disease Control and Prevention field staff.

To find these data, we searched government Web sites using these keywords: case definition, suspect case, confirmed case, COVID-19, case criteria, surveillance, testing criteria, guidelines, laboratory, reverse transcription PCR (RT-PCR), and asymptomatic. All surveillance definitions and testing criteria were verified current as of January 1, 2021. Several official policies were not available in English. For these documents, we used Google Translate (https://translate.google.com) to identify definitions and testing policies.

### Data Management and Analysis

To compare case definitions across countries, we classified the components of each definition into 3 parts: diagnostic components including a laboratory test or radiographic imagery; clinical signs and symptoms, such as cough, fever, and severe acute respiratory infection; and epidemiologic criteria, including travel to a high-burden region or contact with a confirmed or suspected case. For each country’s testing policy, we reviewed which persons were eligible for diagnostic testing. Countries were classified as testing asymptomatic persons without any additional criteria; testing asymptomatic persons with some epidemiologic criteria, such as contact with a confirmed case; or recommending testing exclusively for symptomatic persons. These analyses were based solely on diagnostic testing eligibility criteria and did not consider exceptions, such as testing asymptomatic persons before travel, asymptomatic testing through the private sector, or local-level mass testing. Exceptions to national testing policies varied on a local level and frequently changed, which made data difficult to procure and unreliable. We compared elements of national case definitions and testing criteria against global norms from WHO.

### Source Assessment

To assess sources, we extracted information on their origin (government source or personal communication) and timeliness (date of publication). We compiled the date of publication and presumed implementation of each country’s most recent case definition as verified on January 1, 2021 (Appendix Table 1). Publication dates range from March 27, 2020, to December 18, 2020, for case definitions and July 6, 2020, to January 1, 2021, for testing policies.

## Results

### Suspected Case Definitions

We identified suspected case definitions in 24 (96%) of 25 countries (Table 1; Appendix Tables 2, 3). Although Israel does not have an official suspected case definition, persons are considered suspected on the basis of contact with confirmed cases, which is determined by digital surveillance of cellphones. We interpreted Israel's suspected contact determined by cellphones to be an epidemiologic criterion. The 3 most common criteria in suspected case definitions were fever (reported in 92% of countries), cough (reported in 84% of countries), and labored breathing (reported in 84% of countries). In 7 countries (28%), other criteria were used in addition to common criteria (Table 1). The WHO suspected case definition relies on clinical symptoms, including the 3 most common, and epidemiologic criteria. A total of 14 (56%) countries followed this guidance broadly by using clinical and epidemiologic criteria, 10 (40%) countries required clinical symptoms alone for the suspected case definition, and 2 countries (8%) also incorporated diagnostic testing. The United States relies on laboratory evidence, including antibody or antigen positivity, without any clinical symptoms or epidemiologic criteria, whereas Colombia primarily relies on epidemiologic criteria and clinical symptoms but includes laboratory and radiologic tests as part of their definition to assist with diagnoses (*17*,*18*).

**Table 1:** Selected suspected case definition criteria across 25 countries with the highest COVID-19 case counts, current as of January 1, 2021*

Country	Diagnostic testing/laboratory evidence†	Clinical symptoms								Epidemiologic criteria, any
		Cough	Fever	SARI	Labored breathing	Headache	Muscle pain	Loss of taste or smell	Diarrhea	
WHO definition (reference)		X	X	X	X	X	X		X	X
Argentina		X	X	X	X	X	X	X	X	X
Bangladesh‡		X	X	X	X	X	X		X	X
Brazil		X	X	X	X	X		X	X	
Chile		X	X	X	X	X	X	X	X	X
Colombia	X	X	X		X	X	X	X	X	X
France§¶		X	X		X	X	X	X	X	
Germany§¶		X	X		X	X	X	X	X	
India#		X	X	X	X					X
Indonesia			X	X	X					X
Iran			X	X						X
Iraq‡		X	X	X	X	X	X		X	X
Israel**										X
Italy§¶		X	X		X	X	X	X	X	
Mexico		X	X		X	X	X	X		
Pakistan#		X	X	X	X					X
Peru		X	X	X	X	X				
Philippines‡		X	X	X	X	X	X		X	X
Russia		X	X		X		X	X	X	
Saudi Arabia		X	X	X	X	X		X	X	X
South Africa		X	X	X	X		X	X	X	X
Spain		X	X	X	X	X	X	X	X	
Turkey		X	X	X	X	X	X	X	X	X
Ukraine		X	X	X	X					X
United Kingdom¶		X	X					X		
United States	X									
No. (%) countries including criterion††	2 (8)	21 (84)	23 (92)	16 (64)	21 (84)	15 (60)	14 (56)	14 (56)	15 (60)	16 (64)

*Complete data are available in Appendix Table 3. X indicates the criterion was sufficient for, or a potential component of, the suspected case definition requirement(s). COVID-19, coronavirus disease; SARI, severe acute respiratory infection; WHO, World Health Organization. †See suspected case definition for applicable country (Appendix Table 2). ‡World Health Organization definition (updated August 2020). §European Centre for Disease Prevention and Control definition. ¶These countries consider these definitions as possible not suspected cases; because of the comparability between possible and suspected, we treated these definitions as a suspected definition. #World Health Organization definition (updated March 2020). **Israel does not have an official suspect case definitions persons are considered suspected on the basis of contact with confirmed cases determined by digital surveillance of cellphones. ††Denominator is 24 countries with suspected case definition.

### Probable Case Definitions

We identified probable case definitions in 16 (64%) of 25 countries (Table 2; Appendix Tables 4, 5). The remaining 9 (36%) countries chose not to use a probable case definition and instead use only suspected and confirmed case definitions. The WHO probable case definition includes criteria from all 3 categories: diagnostic testing (chest imaging), clinical symptoms, and epidemiologic criteria. Of the 16 countries, 12 (75%) were consistent with WHO and included criteria from all 3 categories. The number of required criteria across countries was heterogeneous. The 3 most common criteria in probable case definitions were fever (reported in 94% of 16 countries), labored breathing (reported in 88% of 16 countries), and confirmed contact with a probable or confirmed case (reported in 81% of 16 countries). Fourteen (88%) countries included diagnostic testing for the probable case definition, 15 (94%) included clinical symptoms in their definitions, and 14 (88%) included epidemiologic criteria.

**Table 2:** Selected probable case definition criteria across 25 countries with the highest COVID-19 case counts, current as of January 1, 2021*

Country	Diagnostic testing				Clinical symptoms						Epidemiologic criteria		
Inconcl. test	Antigen test	Radiograph imaging	Cough	Fever	SARI	Labored breathing	Loss of taste or smell	Travel history	Hosp.	Confirmed contact
WHO definition (reference)			X		X	X	X	X	X		X	X	X
Argentina													
Bangladesh†			X		X	X	X	X	X		X	X	X
Brazil													
Chile	X	X	X		X	X	X	X	X			X	X
Colombia	X				X	X		X	X		X		X
France‡			X		X	X		X	X				X
Germany‡			X		X	X		X	X				X
India§	X				X	X	X	X			X	X	X
Indonesia						X	X	X					
Iran			X			X	X		X		X		X
Iraq†			X		X	X	X	X	X		X	X	X
Israel													
Italy‡			X		X	X		X	X				X
Mexico													
Pakistan§	X				X	X	X	X			X	X	X
Peru													
Philippines†			X		X	X	X	X	X		X	X	X
Russia					X	X	X	X	X		X		X
Saudi Arabia													
South Africa													
Spain	X		X		X	X	X	X	X				
Turkey													
Ukraine	X												
United Kingdom													
United States		X	X		X	X	X	X	X				X
No. (%) countries including criterion¶	6 (38)	2 (13)	10 (63)		13 (81)	15 (94)	11 (69)	14 (88)	12 (75)		8 (50)	6 (38)	13 (81)

*Complete data are available in Appendix Table 5; full probable case definitions are shown in Appendix Table 4. X indicates the criterion was sufficient for, or a potential component of, the probable case definition requirement(s). COVID-19, coronavirus disease; hosp., hospitalized; inconcl., inconclusive; SARI, severe acute respiratory infection; WHO, World Health Organization. †World Health Organization definition (updated August 2020). ‡European Centre for Disease Prevention and Control definition. §World Health Organization definition (updated March 2020). ¶Denominator is 16 countries with probable case definition.

### Confirmed Case Definitions

We identified confirmed case definitions in all 25 countries (100%) (Table 3; Appendix Tables 6, 7). All confirmed case definitions required diagnostic testing. A total of 18 (72%) countries were consistent with WHO’s recommendations and specified RT-PCR tests in their case definition. Of these countries, 10 (40%) also included antigen or antibody tests in their definition. In 7 (28%) countries, the type of diagnostic test was not specified. Reference to the suspected case definition within the confirmed case definition was included in 7 (28%) of countries. Of these, Mexico, Saudi Arabia, and Turkey required that a person meet the suspected case definition in addition to diagnostic testing criteria. In addition to confirming cases on the basis of diagnostic testing, 6 (24%) countries confirmed cases exclusively on the basis of loss of taste or smell (anosmia or ageusia). Overall, 8 countries (32%) included clinical symptoms as part of their confirmed case definition.

**Table 3:** Selected confirmed case definition criteria across 25 countries with the highest COVID-19 case counts, current as of January 1, 2021*

Country	Diagnostic testing						Clinical symptoms			Epidemiologic criteria		
PCR test	Antigen test	Ab test	Positive test (NS)	Radiograph imaging	Meet suspected case definition	Loss of taste or smell	Travel history	Hosp.	Confirmed contact
WHO definition (reference)	X											
Argentina	X	X					X	X				X
Bangladesh†	X											
Brazil	X	X	X		X			X				X
Chile	X						X	X			X	
Colombia	X	X										
France‡	X	X										
Germany‡	X	X										
India†				X								
Indonesia	X											
Iran				X								
Iraq†	X											
Israel				X								
Italy‡	X	X										
Mexico				X			X	X				X
Pakistan †				X								
Peru	X	X					X			X	X	X
Philippines†	X	X										
Russia	X	X	X									
Saudi Arabia	X						X			X	X	X
South Africa	X											
Spain	X	X					X	X				
Turkey	X						X	X		X	X	X
Ukraine				X								
United Kingdom				X								
United States	X											
No. (%) countries§	18 (72)	10 (40)	2 (8)	7 (28)	1 (4)		7 (28)	6 (24)		3 (12)	4 (16)	6 (24)

*Complete data are available in Appendix Table 7 ; X indicates the criterion was sufficient for, or a potential component of, the confirmed case definition requirement(s). Full confirmed case definitions can be found in Appendix Table 6. Ab, antibody; hosp., hospitalized; NS, not specified; WHO, World Health Organization. †World Health Organization definition (confirmed case definition did not change between March 2020 and August 2020 update). ‡European Centre for Disease Prevention and Control definition. §Denominator is 25 countries with confirmed case definitions.

### Testing Eligibility Criteria

We identified testing criteria in all 25 countries (100%) (Appendix Table 8). Of those, 8 (32%) countries had no symptom requirements for testing, 8 (32%) had no symptom requirements for testing but required epidemiologic criteria (i.e., exposure to a confirmed or probable case), and 9 (36%) countries required symptoms. Of the 8 countries requiring epidemiologic criteria, 5 (63%) also allowed testing for asymptomatic healthcare workers (Appendix Table 8). Policies from Saudi Arabia and the United Kingdom specified not to test asymptomatic persons but included an exception for healthcare workers (Appendix Table 8). WHO recommends testing asymptomatic persons who have had contact with a confirmed case; 64% of countries used eligibility criteria at least as permissive as WHO.

### Source Assessment

We found 92% of case definitions on government Web sites, and 72% were published or included in documents published after the most recent WHO definition was published (August 7, 2020) (Appendix Table 1). India and Pakistan used the previous WHO definition (dated March 2020); we could not confirm that these countries updated their definition on the basis of the newest WHO definitions. We could not locate definitions on government Web sites for Israel, Iraq, and Iran; for these countries, we obtained definitions from personnel involved in the country’s COVID-19 response. Of 25 countries, 23 (92%) had an official government source for diagnostic testing criteria. In total, 88% of testing criteria were published after September 1, 2020. The policies for Philippines, Brazil, and Pakistan were updated in July and August 2020.

## Discussion

All iterations of WHO’s global COVID-19 surveillance guidance state that countries might need to adapt case definitions to their specific circumstances (*5*–*7*,*19*). Beginning with the March 20, 2020, version, WHO also encouraged countries to publish their adapted versions online and in periodic situation reports (*6*,*7*). Nearly all countries (92%) in this analysis chose to deviate from WHO case definitions; 92% of countries posted their case definition on a government Web site. Suspected and confirmed case classifications were found for nearly all countries, but 36% excluded the probable case classification. In addition, we observed substantial variation among testing criteria used in national case definitions. Although WHO reserved the use of laboratory testing for confirmed cases only, 2 (8%) countries included laboratory evidence for suspected cases and 14 (88%) for probable cases; 32% included nonlaboratory criteria for confirmed cases. Laboratory evidence in some countries was not restricted to RT-PCR and included increasingly available antigen and antibody tests. Testing eligibility criteria also differed widely; many countries either excluded asymptomatic persons from routine testing (36%) or only included them under certain conditions (32%).

Differences in case definitions and testing eligibility can affect efforts to monitor disease trends and determine the impact and effectiveness of vaccines across countries and over different periods. As knowledge of a novel disease increases, the sensitivity and specificity of the case definition changes over time, ultimately affecting the number of cases identified (*20*). For example, during the 2002–2003 severe acute respiratory syndrome outbreak, several iterations of case definitions in the Netherlands diverged from the more sensitive and less specific WHO case definition. When all cases were reevaluated, 21 cases were classified as suspected and 2 as probable according to the latest WHO case definition, as opposed to 9 suspected and zero probable cases according to the Netherlands case definitions (*21*). As the COVID-19 pandemic emerged in China, a February 12, 2020, change to the case definition to include clinically diagnosed mild cases resulted in identification of >15,000 cases (*22*). A study of successive case definitions in China, each with gradually increasing sensitivity, also yielded higher detection of cases (*15*). During January 15–March 3, 2020, the National Health Commission of China used 7 versions of the case definition for COVID-19; the study estimates that the proportion of cases detected increased by 7 times after the first change, 3 times from change 2 to 4, and 4 times after change 5. The authors estimated that if the fifth version of the case definition had been applied throughout the outbreak, 4.2 times more confirmed cases would have been identified in China by February 20, 2020 (232,000 vs. 55,508). A more recent study benefitting from the availability of the complete genome sequence for SARS-CoV-2 and access to respiratory specimens collected early for retrospective analysis perhaps best demonstrates the effect of a restrictive case definition (*23*). Those authors identified multiple early cases of SARS-CoV-2 infection in Nottingham, UK, that, despite demonstrating symptoms consistent with COVID-19, did not meet case definition criteria used for diagnostic testing referral in place at the time because of lack of travel history or contact with an infected person. Genomic sequencing of these undetected cases revealed that most were acquired locally by community spread before widespread mitigation measures were adopted. These findings suggest that countries that used less sensitive case definitions, particularly at the start of the pandemic, might have grossly underestimated the true burden of disease, which affected decisions about the need for and timing of infection control measures. Changes in case definitions, both across and within countries, might also need to be considered when analyzing an epidemic curve for COVID-19 or other novel diseases.

The wide variation we found in suspected and probable case criteria and the complete omission of the probable case classification in some nations is of particular interest. WHO indicated that suspected and probable case definitions were revised to reflect increased knowledge of the clinical spectrum of COVID-19 signs and symptoms, especially the most common and predictive. These updates informed global and national surveillance because some symptoms have limited predictive value for surveillance purposes despite their frequent inclusion in case identification procedures (*24*–*27*). In its August 7, 2020, guidance, WHO delineated recommendations for handling each case classification. These recommendations included investigating suspected and probable cases for the presence of SARS-CoV-2 by using available laboratory tests, conducting contact tracing with persons with eligible exposure to probable and confirmed cases, and providing specific types of notifications within 24 hours of identifying probable and confirmed cases (*8*). This guidance also included a new request for countries to include counts of probable cases and confirmed cases in weekly aggregate reports.

WHO case definition guidance does not explicitly state a type of test for diagnostic confirmatory testing but references laboratory guidance that recommend nucleic acid amplification tests (NAATs), such as RT-PCR (*28*). Many countries might not have considered the laboratory guidance and used the WHO confirmed case definition verbatim. Indeed, we found that 7 countries did not specify a type of test for confirmatory testing. Results indicating some countries’ use of alternatives to NAAT as laboratory evidence is another key finding. Antigen tests, particularly point-of-care tests, have been promoted as a tool for early detection and preventing asymptomatic spread (*10*). However, their sensitivity is generally lower than NAATs, leading to false negatives (*29*). Antibody tests have typically been recommended as a surveillance assay rather than a standalone diagnostic tool (*12*,*30*). Despite the limitations of NAAT alternatives, their increasing availability in many areas and benefits such as lower overall cost, simplified logistics and supply chain management, and faster turnaround of results for rapid versions could explain their integration in some confirmed case definitions.

In all 25 countries, confirmed cases relied on diagnostic testing. Characterizing differences in eligibility for diagnostic testing across countries helps determine whether different persons are being diagnosed and designated as a confirmed case. For example, a country that requires symptoms and epidemiologic transmission to be eligible for diagnostic testing might have fewer cases detected than if they permitted testing to all persons in a country regardless of clinical or epidemiologic criteria. Early testing strategies targeted segments of the population believed to be at greatest risk for exposure to SARS-CoV-2. For example, national testing policy in Australia emphasized defining and targeting high-risk settings, such as residential care facilities or correctional facilities (*31*). In May 2020, the European Centre for Disease Prevention and Control expanded the pool of persons eligible for laboratory testing, resources permitting, to include asymptomatic persons in healthcare settings and long-term care facilities to identify potential sources of infection and protect vulnerable persons (*14*). WHO recommendations for laboratory testing also evolved over time and acknowledged that testing priorities would depend on intensity of transmission, number of cases, and laboratory capacity. On June 25, 2021, WHO released updated guidance that called on member states to create a national testing strategy that adapts to these changes and to implement public health actions that break transmission chains (*32*). Specific strategies for different SARS-CoV-2 transmission scenarios might include testing more persons than those who meet the latest suspected and probable case definitions, such as patients with unexpected clinical manifestations, asymptomatic contacts, and samples from existing sentinel surveillance sites. In addition, the guidance includes alternative testing strategies when laboratory capacity is low or overstretched.

Because of the large proportion of asymptomatic or mildly symptomatic COVID-19 cases, detecting both symptomatic and asymptomatic cases is necessary to ensure accurate case counts (*33*). Including asymptomatic cases also affects key epidemiologic metrics, such as incidence and case-fatality ratio. Although expansive testing criteria would increase the likelihood of detecting asymptomatic infections, this benefit should be weighed against the effect tracing and testing these eligible persons would have on the public health system (*31*). For example, broadening testing eligibility criteria might overload the healthcare system with persons who have low probability of infection or disease progression. Furthermore, many settings might not have adequate resources to test all eligible persons (*33*). Although WHO provides harmonious global testing criteria and case definitions, our findings suggest heterogeneity in how these aspects were adapted; it might be necessary to account for these deviations when comparing and collating COVID-19 case counts across countries.

The first limitation of our study is that, although the included studies represented ≈85% of reported cases globally, case definitions in the countries making up the remaining 15% of cases might differ. In addition, we chose to include the 25 countries with the highest number of reported cases, which might not represent the countries with the highest number of infections. Including countries on the basis of new infections from population-based serosurveillance or other sources merits future research. Second, although we identified suspected case definitions, confirmed case definitions, and testing criteria for most countries, we identified probable case definitions in only 16. This difference could be because of the lack of a probable case definition or its lack of availability in the public domain; regardless, the results of the probable case definition analyses might be less generalizable than the others. Third, we used Google Translate to translate definitions not in English in lieu of direct translation by native or bilingual speakers. Previous studies have used Google Translate for health-related text, including an analysis of national health agency mask guidance across multiple countries and regions during the COVID-19 pandemic (*34*). One study specifically compared the agreement between translations of abstracted data from published clinical trials between native speakers and Google Translate for 9 different languages and determined that agreement ranged from 85% to 97% (*35*). In our study, translation errors could have occurred for some languages and thus created discrepancies between the original policy intent and our interpretation of the translation. Fourth, our scope was limited to confirmed, probable, and suspected case definitions; other classifications, such as persons under investigation, might merit further research. Fifth, after extraction and analyses were completed, additional issues relating to case surveillance have emerged. These issues include cases amongst vaccinated persons, criteria for distinguishing a new case from an existing case (i.e., reinfection cases), as well as variants (*17*,*36*–*39*). Although these issues were not part of national case surveillance definitions, case surveillance amongst vaccinated persons can help inform stakeholders of changes in vaccine effectiveness, reinfection surveillance might provide further information on naturally acquired and vaccine-acquired immunity, whereas genomic surveillance could provide further insights on circulating strains. All three elements are vital to comprehensive national surveillance of COVID-19. Sixth, given the large number of possibilities, we chose not to list every permutation of laboratory, clinical, and epidemiologic criteria for WHO and national suspected, probable, and confirmed case definitions. Finally, despite our analysis of each government’s policies, these policies might not be implemented equally in various settings and could change over time. To continue to build on the implications of this study, further research should determine the programmatic implications of less sensitive case definitions, such as whether misclassifying cases leads to outbreaks and onward population transmission.

Case surveillance remains the foundation for national COVID-19 surveillance and plays a vital role in ongoing situational awareness, clarifying the impact and effectiveness of vaccines and informing other public health and social measures. We observed marked heterogeneity in testing eligibility requirements among countries and how countries define COVID-19 cases. Specifically, we observed heterogeneity in eligible clinical symptoms for suspected case definitions, laboratory and diagnostic requirements for probable case definitions, and eligible laboratory assays for confirmed case definitions. Testing eligibility criteria varied from being restricted to populations with exposure and symptoms to all populations being eligible, regardless of exposure and symptoms. Collectively, these issues suggest that efforts to compare and collate COVID-19 case counts across countries require careful interpretation. Improved harmonization of case definitions across countries prospectively for COVID-19, and for other novel infectious diseases that might emerge, warrants consideration.

This article was preprinted at https://www.medrxiv.org/content/10.1101/2021.05.11.257047v1.

Appendix Additional information about coronavirus disease case definitions, diagnostic testing criteria, and surveillance in 25 countries with highest reported case counts
