# Supplementary material for: Coronavirus Disease Case Definitions, Diagnostic Testing Criteria, and Surveillance in 25 Countries with Highest Reported Case Counts
Source: Emerg Infect Dis. 2022 Jan;28(1):148–56. doi: 10.3201/eid2801.211082 (PMC8714223; doi:10.3201/eid2801.211082)
Supplement: Appendix — Additional information about coronavirus disease case definitions, diagnostic testing criteria, and surveillance in 25 countries with highest reported case counts [file 21-1082-Techapp-s1.pdf]

Article DOI: https://doi.org/10.3201/eid2801.211082

Coronavirus Disease Case Definitions, Diagnostic Testing Criteria, and Surveillance in 25 Countries with Highest Reported Case Counts

Appendix

Appendix Table 1. Assessment of sources for COVID-19 case definition and testing criteria across 25 countries with the highest case counts, current as of January 1, 2021*

Countries	Case definition source (date)†	Testing criteria source (date)†
WHO	WHO (*1*) (2020 Aug 7)	NA
Argentina	Government (*2*) (2020 Sep 11)	Government (*3*) (2020 Sep 23)
Bangladesh	Government (*4*) (2020 Nov 5)	Government (2020 Nov 5)
Brazil	Government (*5*) (2020 Aug 5)	Government (2020 Aug 5)
Chile	Government (*6*) (2020 Oct 1)	Government (*7*) (2020 Nov 18)‡
Colombia	Government (*8*) (2020 Oct 19)	Government (*9*) (2020 Oct)
France	ECDC (*10*) (2020 Dec 3)	Government (*11*) (2020 Oct 19)‡
Germany	ECDC (*10*) (2020 Dec 3)	Government (*12*) (2020 Dec 16)
India	Government (*13*) (2020 Jul3)	Government (*14*) (2020 Sep 4)
Indonesia	Government (*15*) (2020 Jul 13)	Government (*16*) (2021 Jan 1)§
Iran	CDC contact (N. Farag, CDC, pers. comm., 2020 Sep 29)†	CDC contact (N. Farag, CDC, pers. comm., 2020 Sep 29)†
Iraq	CDC contact (Y.Y. Majeed, CDC, pers. comm., 2020 Oct 2)†	CDC contact (Y.Y. Majeed, CDC, pers. comm., 2020 Oct 2)†
Israel	CDC contact (H. Burke, CDC, pers. comm., 2020 Oct 7)†	Government (17) (2020 Dec 17)
Italy	ECDC (*10*) (2020 Dec 3)	Government (18) (2020 Oct 23)
Mexico	Government (*19,20*) (2020 Aug 25)	Government (*21*) (2020 Nov 11)
Pakistan	Government (*22*) (2020 Mar 27)	Government (*23,24*) (2020 Dec 2)
Peru	Government (*25*) (2020 Jul 10)	Government (*26*) (2020 Sep 30)
Philippines	Government (27) (2020 Nov 25)	Government (28) (2020 Jul 6)
Russia	Government (29) (2020 Oct 26)	Government (*30*) (2021 Jan 1)§**
Saudi Arabia	Government (*31*) (2020 Oct)	Government (*31*) (2020 Oct)
South Africa	Government (*32*) (2020 Aug 18)	Government (*33*) (2020 Sep 16)
Spain	Government (*34*) (2020 Dec 18)	Government (*34*) (2020 Dec 18)
Turkey	Government (*35*) (2020 Dec 7)	Government (*35*) (2020 Dec 7)
Ukraine	Government (*36*) (2020 Mar 28)	Government (37) (2021 Jan 1)§**
United Kingdom	Government (38) (2020 Sep 28)	Government (39) (2021 Jan 1)§**
United States	Government (*40*) (2020 Aug 5)	Government (*41*) (2020 Oct 21)

*CDC, Centers for Disease Control and Prevention; ECDC, European Centre for Disease Prevention and Control; NA, not applicable; WHO, World Health Organization.
†Date of Centers for Disease Control and Prevention contact communication.
‡Date the website was last updated.
§Date the source website was last verified in absence of website update date.

Appendix Table 2. Full suspected case definitions of COVID-19 in countries with highest reported case counts*

Country	Definition
WHO (reference)<br>Iraq†<br>Bangladesh†<br>Philippines†	1. A person who meets the clinical AND epidemiologic criteria: Clinical Criteria: Acute onset of fever AND cough; OR Acute onset of ≥3 of the following signs or symptoms: fever, cough, general weakness/fatigue, headache, myalgia, sore throat, coryza, dyspnea, anorexia/nausea/vomiting, diarrhea, altered mental status. Epidemiologic Criteria: Residing or working in an area with high risk for transmission of virus such as closed residential settings or humanitarian settings such as camp and camp-like settings for displaced persons, anytime within the 14 days before symptom onset; OR Residing or travel to an area with community transmission anytime within the 14 days before symptom onset; OR Working in any healthcare setting, including within health facilities or within the community, anytime within the 14 days before symptom onset. 2. A patient with severe acute respiratory illness: acute respiratory infection with history of fever or measured fever of ≥38°C and cough, with onset within the last 10 days and requiring hospitalization.
India<br>Pakistan	1. A patient with acute respiratory illness (fever and ≥1 sign/symptom of respiratory disease, such as cough or shortness of breath) AND a history of travel to or residence in a location reporting community transmission of COVID-19 during the 14 days before symptom onset. 2. A patient with any acute respiratory illness AND having been in contact with a confirmed or probable person with COVID-19 (see definition of contact) in the 14 days before symptom onset. 3. A patient with severe acute respiratory illness (fever and ≥1 sign/symptom of respiratory disease, such as cough or shortness of breath AND requiring hospitalization) AND in the absence of an alternative diagnosis that fully explains the clinical manifestation.
United States	Meets supportive laboratory evidence with no previous history of being a confirmed or probable case. Supportive laboratory evidence: Detection of specific antibody in serum, plasma, or whole blood. Detection of specific antigen by immunocytochemistry in an autopsy specimen.
Italy‡§<br>Germany‡§<br>France‡§	Any person meeting clinical criteria: ≥1 of the following symptoms: cough, fever, shortness of breath, or sudden onset of anosmia, ageusia, or dysgeusia. Additional less specific symptoms include headache, chills, muscle pain, fatigue, vomiting, or diarrhea.
Brazil	1.Individual with acute respiratory condition, characterized by at ≥2 of the following signs and symptoms: fever (even if referred), chills, sore throat, headache, cough, runny nose, olfactory disorders or taste disorders. In children, nasal obstruction is also considered in addition to the previous symptoms, in the absence of another specific diagnosis. In the elderly, specific aggravation criteria such as syncope, mental confusion, excessive sleepiness, irritability, and inappetence should also be considered. If COVID-19 is suspected, fever might be absent and gastrointestinal symptoms (diarrhea) might be present. 2. Individual with Influenza-like illness who has dyspnea or respiratory discomfort OR persistent pressure in the chest OR O_2 saturation <95% in room air OR bluish color of the lips or face. In children, in addition to the previous items, flapping of the nose, cyanosis, intercostal circulation, dehydration, and lack of appetite.
Russia	Clinical manifestations of acute respiratory infection: temperature >37.5°C and ≥1 of the following: cough (dry or with scanty sputum), shortness of breath, feeling of congestion in the chest, satiety blood oxygen according to pulse oximetry (SpO2) ≤95%, sore throat, nasal congestion or mild rhinorrhea, impaired or loss of smell (hyposmia or anosmia), loss of taste (dysgeusia), conjunctivitis, weakness, muscle pain, headache, vomiting, diarrhea, or skin rash in the absence of other known causes, which explain the clinical picture regardless of the epidemiologic anamnesis.
Colombia	Person with exposure because of having been in a place with community transmission or outbreak or contact with probable cases, and with respiratory OR nonrespiratory clinical manifestations of COVID-19 of any severity, clinical laboratory or radiologic findings belonging to groups of risk factors or vulnerability. Also, asymptomatic persons with exposure to probable or confirmed COVID-19 case-patients. Respiratory clinical manifestations: fever >38°C, cough, fatigue, expectoration, shortness of breath or dyspnea, sore throat, rhinorrhea, Sp02 <93%. Nonrespiratory clinical manifestations: anosmia, hyposmia, ageusia, dysgeusia, diarrhea, anorexia, nausea and vomiting, abdominal pain or discomfort, acute conjunctivitis, seizures, vertigo, headache, myalgia, skeletal muscle injuries, altered consciousness, acute cerebrovascular disease, ataxia, seizures, meningoencephalitis, Guillain-Barre syndrome, mental status disorders, hepatic compromise due to elevated aminotransferases, erythematous rash, hive rash, vesicles, acral ischemia, unilateral transient livedo reticularis, acute cardiac injury, heart failure arrhythmia, shock, acute myocarditis, chest tightness, acute kidney injury, urinary symptoms or cystitis, coagulation disorders, thrombotic events, antiphospholipid antibodies, hearing loss, or hearing discomfort. Clinical laboratory and radiological findings: laboratory and nonetiologic clinical diagnostic tests: Albumin Decrease (81%), CRP increase (79%) (MIS-C 94%), LDH increase (69.3%), Thrombocytosis (61%), Lymphopenia (57.5%, 25%, 56.5%), Interleukin-6 increase (56%), AST increase (37%), Leukopenia (28%, 25%), Leukocytosis (18.3%), Neutrophilia (MIS-C 83%), Thrombocytopenia (13%, 16.4%–32.3%), Increase in D-dimer, Ferritin, Decrease in T3 and T 4 Troponin T (MIS-C 68%), pro BNP (MIS-C 77%). Pulmonary radiological findings Bilateral commitment (81%), Consolidation (73.5%), Ground Glass Opacity (73.5%), Abnormal echocardiogram (MIS-C 59%, 63%). Factors of vulnerability: healthcare workers, workers who serve in high volumes of public work in social protection, workers with high mobility because of occupational activity, and internal Colombian migrants.
Peru	Person with acute respiratory infection experiencing cough or pain from throat and ≥1 of the following signs / symptoms: general discomfort, fever, headache, difficulty breathing, nasal congestion.
Argentina	1. Anyone (of any age) who has ≥2 of the following symptoms: fever (≥37.5°C), cough, odynophagia, shortness of breath, headache, myalgia, diarrhea, or vomiting, with no other etiology that fully explains the clinical manifestation. This criterion includes any severe acute respiratory infection. 2. Sudden loss of taste or smell, in the absence of any other identified cause. 3. Anyone who is a healthcare worker, resides or works in closed or long-term institutions, is essential personnel, lives in popular neighborhoods or native towns, or had close

Country	Definition
	contact with confirmed a COVID-19 case-patient within 14 days of contact AND has >1 of these symptoms: fever (37.5°C or higher), cough, odynophagia, shortness of breath, or sudden loss of taste or smell.
Spain	Anyone with a clinical picture of acute respiratory infection of sudden onset of any severity that occurs, among others, with fever, cough, or shortness of breath. Other symptoms such as odynophagia, anosmia, ageusia, muscle pain, diarrhea, chest pain, or headache, among others, might also be considered symptoms of suspected SARS-CoV-2 infection according to clinical judgment.
Mexico	Person of any age who has had >1 of the following signs and symptoms in the last 10 days: cough, fever, dyspnea (serious condition), or headache. In children <5 years of age, irritability can replace headache. Accompanied by >1 of the following minor signs or symptoms: myalgia, arthralgias, odynophagia, chills, chest pain, rhinorrhea, anosmia, dysgeusia, or conjunctivitis.
South Africa	Any person presenting with an acute (<10 days) respiratory tract infection or other clinical illness compatible with COVID-19, or an asymptomatic person who is a close contact a of a confirmed case-patient. Symptoms include ANY of the following respiratory symptoms: cough, sore throat, shortness of breath, anosmia (loss of sense of smell), or dysgeusia (alteration of the sense of taste), with or without other symptoms (which might include fever, weakness, myalgia, or diarrhea).
United Kingdom‡	New continuous cough or temperature >37.8°C or loss of or change in normal sense of smell (anosmia) or taste (ageusia).
Iran	1. Acute onset of fever OR acute onset of ILI/ARI symptoms or nausea and vomiting AND epidemiologic criteria (residing/working/travel to high-risk settings or community transmission or health facilities within 14 days of symptom onset). 2. Severe acute respiratory infection.
Chile	1. Patient presenting an acute picture with >2 of the symptoms compatible with COVID-19: fever (>37.8°C), cough, dyspnea, chest pain, odynophagia, myalgia, chills, headache, diarrhea, or sudden loss or decrease of smell (anosmia or hyposmia) or taste (ageusia or dysgeusia). 2. Patient with severe acute respiratory infection requiring hospitalization.
Saudi Arabia	1. Patient with acute respiratory illness (sudden onset of >1 of the following: fever (measured or by history), cough, or shortness of breath. 2. Patient with sudden onset of >1 of the following: headache, sore throat, rhinorrhea, nausea, diarrhea, or loss of smell or taste AND in the 14 days before symptom onset, met >1 of the following criteria: contact with a confirmed COVID-19 case Or Working in or attended a healthcare facility where patients with confirmed COVID-19 were admitted. 3. Any admitted adult patient with unexplained severe acute respiratory illness (SARI), either community-acquired pneumonia or hospital-acquired pneumonia.
Turkey‡	1. At least 1 of the following signs and symptoms: fever, cough, shortness of breath, sore throat, headache, muscle aches, loss of taste and smell, diarrhea AND the clinical picture cannot be explained by another cause or disease AND 1 of the following within 14 days before the onset of symptoms: person or close contact has history of being in a high-risk area for the disease or person has confirmed contact with a COVID-19 case-patient. 2. At least 1 of the signs and symptoms of fever and severe acute respiratory infection (SARI) (cough and respiratory distress), requiring hospitalization, and the clinical picture cannot be explained by another cause or disease. SARI: The need for hospitalization because of fever, cough and dyspnea, tachypnea, hypoxemia, hypotension, diffuse radiological findings on lung imaging, and change in consciousness in a patient with acute respiratory tract infection that developed in the last 14 days. 3. Combination of >2 of the following signs and symptoms: fever, cough, shortness of breath, sore throat, headache, muscle aches, loss of taste and smell or diarrhea, and this situation cannot be explained by another cause or disease.
Indonesia	1. A person who has 1 of the following criteria: persons with acute respiratory infections (ARI) (namely fever (>38°C) or a history of fever and accompanied by any of the symptoms or signs of respiratory disease such as cough, shortness of breath, sore throat, runny nose, pneumonia from mild to severe AND in the last 14 days before symptoms appeared had a history of travel to or lives in a country or territory of Indonesia reporting local transmissions. 2.People with any of the symptoms or signs of ARI AND in the last 14 days before symptoms develop had a history of contact with a confirmed or probable COVID-19 case. 3. People with severe ARD or severe pneumonia requiring hospitalization AND no other cause based on a convincing clinical picture.
Israel	Israel does not have official surveillance case definitions; persons are considered suspect on the basis of contact with confirmed cases determined by digital surveillance of cellphones.
Ukraine	1. A patient with acute respiratory illness (sudden onset, fever, and >1 of the following symptoms: cough or difficulty breathing), regardless of the need for hospitalization, and who 14 days before the onset of symptoms meets >1 of the following epidemiologic criteria: contact with a confirmed or probable case of COVID-19 or visited or resided in a country or region with local transmission of the virus in the community according to WHO situation reports. 2. Patient with severe acute respiratory disease (body temperature >38°C and >1 of the following symptoms: cough or difficulty breathing) requiring hospitalization and the absence of other reasons that fully explain the clinical picture.

*ARD, acute respiratory distress; ARI, acute respiratory infection; AST, Aspartate Aminotransferase; BNP, B-type natriuretic peptide; COVID-19, coronavirus disease; CRP, C-reactive protein; ILI, influenza-like illness; LDH, Lactate Dehydrogenase; MIS-C, multisystem inflammatory syndrome-children; SARI, severe acute respiratory infection; SARS-CoV-2, severe acute respiratory infection coronavirus 2; SpO2, blood oxygen saturation; WHO, World Health Organization.
†World Health Organization definition.
‡These countries use a possible case definition in place of a suspected case definition.
§European Centre for Disease Prevention and Control definition.

Appendix Table 3. Full suspected COVID-19 case definition criteria across 25 countries with the highest case counts, current as of January 1, 2021*

	Diagnostic testing	Clinical symptoms																		Epidemiologic criteria				Other
Country	Laboratory evidence†	Cough	Fever	SARI	Labored breathing	Headache	Muscle pain	Sore throat or odynophagia	Chest pain or congestion	Runny nose or nasal congestion	Loss of taste or smell	Diarrhea	Nausea or vomiting	Joint pain	Chills	Rash	SPO_2 Level	Conjunctivitis	Fatigue or weakness	Travel history	Hospitalized	Confirmed contact	Healthcare employee	Diagnostic, clinical, or EPI†
WHO definition‡ (reference)		X	X	X	X	X	X	X		X		X	X						X	X	X		X	X
Argentina		X	X	X	X	X	X	X			X	X	X									X	X	X
Bangladesh‡		X	X	X	X	X	X	X		X		X	X						X	X	X		X	X
Brazil		X	X	X	X	X		X		X	X	X			X		X							X
Chile		X	X	X	X	X	X	X	X		X	X			X						X			
Colombia	X	X	X		X	X	X	X		X	X	X	X	X		X	X	X	X	X		X	X	X
France§¶		X	X		X	X	X				X	X	X		X				X					
Germany§¶		X	X		X	X	X				X	X	X		X				X					
India#		X	X	X	X															X	X	X		
Indonesia			X	X	X			X		X										X	X	X		
Iran			X	X									X							X				
Iraq‡		X	X	X	X	X	X	X		X		X	X						X	X	X		X	X
Israel**																				X		X		
Italy§¶		X	X		X	X	X				X	X	X		X				X					
Mexico		X	X		X	X	X		X	X	X			X	X			X	X					
Pakistan#		X	X	X	X															X	X	X		
Peru		X	X	X	X	X				X														
Philippines‡		X	X	X	X	X	X	X		X		X	X						X	X	X		X	X
Russia		X	X		X		X	X	X	X	X	X	X			X	X	X	X					
Saudi Arabia		X	X	X	X	X		X		X	X	X	X								X	X	X	X
South Africa		X	X	X	X		X	X			X	X							X			X		
Spain		X	X	X	X	X	X	X	X		X	X												
Turkey		X	X	X	X	X	X				X	X								X	X	X		
Ukraine		X	X	X	X															X	X	X		
United Kingdom¶		X	X								X													
United States	X																							
No. countries including criterion	2	21	23	16	21	15	14	12	4	10	14	15	11	2	6	2	3	3	10	11	10	10	6	7
% of countries††	8%	84%	92%	64%	84%	60%	56%	48%	16%	40%	56%	60%	44%	8%	24%	8%	12%	12%	40%	44%	40%	40%	24%	28%

*X indicates the criterion was sufficient for, or a potential component of, the suspected case definition requirement(s). Full suspected case definitions can be found in Appendix Table 2. SARI, severe acute respiratory infection; SpO_2, blood oxygen saturation; WHO, World Health Organization.
†Refer to suspected case definition for applicable country (Appendix Table 2).
‡World Health Organization definition (updated August 2020).
§European Centre for Disease Prevention and Control definition.
¶These countries consider these definitions as possible not suspected cases; because of the comparability between possible and suspected, we treated these definitions as a suspected definition.
#World Health Organization definition (updated March 2020).
**Israel does not have official surveillance case definitions; persons are considered suspected on the basis of contact with confirmed cases determined by digital surveillance of cellphones.
††Denominator is 24 countries with suspected case definition.

Appendix Table 4. Probable case definitions of COVID-19 in countries with highest reported case counts*

Country	Definition
WHO Definition<br>Iraq†<br>Bangladesh†<br>Philippines†	1. A patient who meets clinical criteria AND is a contact of a probable or confirmed case, or epidemiologically linked to a cluster with ≥1 confirmed case. 2. A suspected case with chest imaging showing findings suggestive of COVID-19. Typical chest imaging findings suggestive of COVID-19 include the following chest radiography: hazy opacities, often rounded in morphology, with peripheral and lower lung distribution; chest CT: multiple bilateral ground glass opacities, often rounded in morphology, with peripheral and lower lung distribution; lung ultrasound: thickened pleural lines, B lines (multifocal, discrete, or confluent), consolidative patterns with or without air bronchograms. 3. A person with recent onset of anosmia (loss of smell) or ageusia (loss of taste) in the absence of any other identified cause. 4. Death, not otherwise explained, in an adult with respiratory distress preceding death AND was a contact of a probable or confirmed case or epidemiologically linked to a cluster with ≥1 confirmed case.
India<br>Pakistan	1. A suspected case for whom testing for the COVID-19 virus is inconclusive. 2. A suspected case for whom testing could not be performed for any reason.
USA	1. Meets clinical criteria AND epidemiologic evidence with no confirmatory laboratory testing performed for COVID-19. 2. Meets presumptive laboratory evidence (detection of SARS-CoV-2 by antigen test in a respiratory specimen). 3. Meets vital records criteria with no confirmatory laboratory evidence. Clinical criteria: ≥2 of the following symptoms: fever (measured or subjective), chills, rigors, myalgia, headache, sore throat, nausea or vomiting, diarrhea, fatigue, congestion, or runny nose OR ≥1 of the following symptoms: cough, shortness of breath, difficulty breathing, new olfactory disorder, new taste disorder OR severe respiratory illness with ≥1 of the following: clinical or radiographic evidence of pneumonia, acute respiratory distress syndrome (ARDS). Epidemiologic criteria: ≥1 of the following exposures in the previous 14 days: close contact with a confirmed or probable case of COVID-19 OR member of a risk cohort as defined by public health authorities during an outbreak.
Italy‡<br>Germany‡<br>France‡	Any person meeting clinical criteria (≥1 of the following symptoms: cough, fever, shortness of breath, sudden onset of anosmia, ageusia or dysgeusia) with epidemiologic link (close contact with confirmed case within 14 days before onset or having been a resident or staff in an institution with ongoing transmission within 14 days before onset) OR any person meeting diagnostic criteria (radiological evidence showing lesions compatible with COVID-19).
Brazil	No probable case definition.
Russia	Same as suspected case AND ≥1 of the epidemiologic signs is present: return from a foreign trip 14 days before the onset of symptoms; having close contact in the last 14 days with a person under monitoring for COVID-19 who subsequently fell ill; having close contact in the last 14 days with a person with laboratory-confirmed diagnosis of COVID-19; availability of professional contacts with persons who have been identified as suspected or confirmed case of COVID-19.
Colombia	Person with any type of individual or multiple exposure to confirmed cases and with respiratory or nonrespiratory clinical manifestations of COVID-19, of any severity, and clinical or radiological laboratory findings. It also includes the person with doubtful etiologic laboratory results (RT-PCR) or laboratory testing is not feasible for some reason. Respiratory clinical manifestations: fever >38°C, cough, fatigue, expectoration, shortness of breath or dyspnea, sore throat, rhinorrhea, Sp02 <93%. Nonrespiratory clinical manifestations: anosmia, hyposmia, ageusia, dysgeusia, diarrhea, anorexia, nausea and vomiting, abdominal pain or discomfort, acute conjunctivitis, seizures, vertigo, headache, myalgia, skeletal muscle injuries, altered consciousness, acute cerebrovascular disease, ataxia, seizures, meningoencephalitis, Guillain-Barre syndrome, mental status disorders, hepatic compromise due to elevated aminotransferases, erythematous rash, hive rash, vesicles, acral ischemia, unilateral transient livedo reticularis, acute cardiac injury, heart failure arrhythmia, shock, acute myocarditis, chest tightness, acute kidney injury, urinary symptoms or cystitis, coagulation disorders, thrombotic events, antiphospholipid antibodies, hearing loss or hearing discomfort.
Peru	No probable case definition.
Argentina	No probable case definition.
Spain	1. Person with severe acute respiratory infection with clinical and radiological symptoms compatible with COVID-19 and negative diagnostic test results, or suspected cases with inconclusive diagnostic test. 2. Cases with high clinical-epidemiologic suspicion with repeatedly negative diagnostic test (≥1 PCR) and positive serologic testing for SARS-CoV-2 performed by high-throughput serologic techniques.
Mexico	No probable case definition.
South Africa	No probable case definition.
United Kingdom	No probable case definition.
Iran	1. Suspected case AND close contact with probable or confirmed case. 2. Suspected case AND positive imaging. 3. Acute onset of anosmia or ageusia without identified cause. 4. Death of a suspected case.
Chile	1. Probable case by laboratory result: patient who meets the definition of a suspected case in whom the PCR result is indeterminate or who has a positive antigenic test for SARS-CoV-2. 2. Probable case because of epidemiologic link: person who has been in close contact with a confirmed case and develops fever (axillary temperature ≈37.8°C) or ≥2 symptoms compatible with COVID-19 within 14 days of contact. The probable case because of an epidemiologic link does not require an RT-PCR test for SARS-CoV-2. If for any reason, a probable case-patient undergoes a confirmatory examination and it is positive, it will be considered as a confirmed case. On the contrary, if the result is negative or indeterminate, it will continue to be considered a probable case. 3. Probable case by imaging: suspicious case with negative RT-PCR result for SARS-CoV-2 but with a chest CT scan with characteristic images of COVID-19 according to the radiological report. 4. Probable case because of symptoms: person with sudden and complete loss of smell (anosmia) or taste (ageusia) without an explanation.

Country	Definition
Saudi Arabia	No probable case definition.
Turkey	No probable case definition.
Philippines	1. Suspected case whose testing for COVID-19 is inconclusive. 2. Suspected case who tested positive for COVID-19 but whose test was not conducted in a national or subnational reference laboratory or officially accredited laboratory for COVID-19 confirmatory testing. 3. Suspected case who died without undergoing any confirmatory testing.
Indonesia	1. Suspected cases with SARI. 2. Died with a convincing clinical picture of COVID-19 AND no RT-PCR laboratory results.
Israel	Israel does not have official surveillance case definitions; persons are considered suspected case-patients on the basis of contact with confirmed cases determined by digital surveillance of cellphones.
Ukraine	A suspected case for whom laboratory testing cannot be unambiguously interpreted.

*COVID-19, coronavirus disease; CT, computed tomography; RT-PCR, reverse transcription PCR; SARI, severe acute respiratory infection; SARS-CoV-2, severe acute respiratory syndrome coronavirus 2.
†World Health Organization definition.
‡European Centre for Disease Prevention and Control definition.

Appendix Table 5. Full probable COVID-19 case definition criteria across 25 countries with the highest case counts, current as of January 1, 2021*

	Diagnostic testing				Clinical symptoms																	Epidemiologic criteria			Other
Country	Inconclusive test	Antigen test	Antibody test	Radiographic imagery	Cough	Fever	SARI	Labored breathing	Headache	Muscle pain	Sore throat or odynophagia	Chest pain or chest congestion	Runny nose or nasal congestion	Loss of taste or smell	Diarrhea	Nausea or vomiting	Chills	Rash	SPO_2 level	Conjunctivitis	Fatigue or weakness	Travel history	Hospitalized	Confirmed contact	Diagnostic, clinical, or EPI†
WHO definition (Reference)				X	X	X	X	X	X	X	X		X	X	X	X					X	X	X	X	X
Argentina																									
Bangladesh‡				X	X	X	X	X	X	X	X		X	X	X	X					X	X	X	X	X
Brazil																									
Chile	X	X		X	X	X	X	X	X	X	X	X		X	X		X						X	X	X
Colombia	X				X	X		X	X	X	X		X	X	X	X		X	X	X	X	X		X	
France§				X	X	X		X						X										X	X
Germany§				X	X	X		X						X										X	X
India¶	X				X	X	X	X														X	X	X	
Indonesia						X	X	X			X		X												X
Iran				X		X	X							X		X						X		X	X
Iraq‡				X	X	X	X	X	X	X	X		X	X	X	X					X	X	X	X	X
Israel																									
Italy§				X	X	X		X						X										X	X
Mexico																									
Pakistan¶	X				X	X	X	X														X	X	X	
Peru																									
Philippines‡				X	X	X	X	X	X	X	X		X	X	X	X					X	X	X	X	X
Russia					X	X	X	X	X	X	X	X	X	X				X	X	X	X	X		X	
Saudi Arabia																									
South Africa																									
Spain	X			X	X	X	X	X	X	X	X	X		X	X										
Turkey																									
Ukraine	X																								
United Kingdom																									
United States		X		X	X	X	X	X	X	X	X		X	X	X	X	X				X			X	X
Country Totals	6	2	0	10	13	15	11	14	8	8	9	3	7	12	7	6	2	2	2	2	6	8	6	13	10
% of countries#	38%	13%	0%	63%	81%	94%	69%	88%	50%	50%	56%	19%	44%	75%	44%	38%	13%	13%	13%	13%	38%	50%	38%	81%	63%

*X indicates the criterion was sufficient for, or a potential component of, the probable case definition requirement(s). Full probable case definitions can be found in Appendix Table 4. SARI, severe acute respiratory infection; SpO_2, blood oxygen saturation; WHO, World Health Organization.
†Refer to probable case definition for applicable country (Appendix Table 4).
‡World Health Organization definition (updated August 2020).
§European Centre for Disease Prevention and Control definition.
¶World Health Organization definition (updated March 2020).
#Denominator is 16 countries with probable case definition.

Appendix Table 6. Confirmed case definitions of COVID-19 in countries with highest reported case counts*

Country	Definition
WHO<br>Iraq†<br>Bangladesh†	A person with laboratory confirmation of 2019-nCoV infection, irrespective of clinical signs and symptoms.
Philippines†	1. Any person, irrespective of presence or absence of clinical signs and symptoms, who was laboratory confirmed for COVID-19 in a test conducted at the national reference laboratory, a subnational reference laboratory, or Department of Health-licensed COVID-19 testing laboratory. 2. Any suspected or probable COVID-19 cases who tested positive using antigen tests in areas without breaks or in remote settings where RT-PCR is not immediately available; provided that the antigen tests satisfy the recommended minimum regulatory, technical, and operational specifications set by the Health Technology Assessment.
India<br>Pakistan	A person with laboratory confirmation of 2019-nCoV infection, irrespective of clinical signs and symptoms.
USA	Meets confirmatory laboratory evidence (detection of SARS-CoV-2 RNA in a clinical specimen using a molecular amplification detection test).
Italy‡<br>Germany‡<br>France‡	Any person meeting laboratory criteria (detection of SARS-CoV-2 nucleic acid or antigen in a clinical specimen).
Brazil	1. Case of ILI or SARI with clinical confirmation associated with anosmia (olfactory dysfunction) OR ageusia (gustatory dysfunction) without any other previous cause. 2. Case of ILI or SARI with history of close or home contact, in the 14 days before appearance of signs and symptoms with confirmed case. 3. Case of ILI or SARI or death due to SARS that could not be confirmed by laboratory criteria AND that presents ≥1 of the following tomographic changes: peripheral, bilateral, frosted glass opacity, with or without consolidation or visible intralobular lines (“paving”), or multifocal matte glass opacity with rounded morphology with or without consolidation or visible intralobular lines (“paving”), or reverse halo sign or other findings of organizing pneumonia (seen later in the disease). 4. Case of ILI or SARI with test of positive result for SARS-CoV-2 performed by the RT-PCR method in real time or REAGENT result for IgM, IgA and / or IgG performed by the following methods: ELISA; immunochromatography (rapid test) for antibody detection; ECLIA; Or Antigen Search: reagent result for SARS-CoV-2 by the Immunochromatography method for antigen detection. 5. Asymptomatic persons with 1 of the following results: positive result for SARS-CoV-2 performed by the RT-PCR method in real time or immunological reagent result for IGM and/or IGA performed by the following methods: ELISA or immunochromatography (rapid test) for antibody detection.
Russia	1. A positive laboratory test result for the presence of RNA SARS-CoV-2 using NAAT or SARS-CoV-2 antigen using immunochromatographic analysis regardless of clinical manifestations.2. Positive result for IgA, IgM and / or IgG antibodies in patients with a clinically confirmed COVID-19 infection.
Colombia	Person with laboratory (RT-PCR for SARS-CoV-2 (MIS-C 59%, 13%–69%) or Antigen detection test (alternative diagnosis. May change according to new evidence)) with positive results of active infection by the SARS-CoV-2 virus regardless of the presence or absence of clinical criteria, since the tests can be performed in asymptomatic, suspected or probable patients, with different prioritization.
Peru	1.Suspected case with a positive laboratory test for COVID 19, be it an RT-PCR test in respiratory samples and/or a rapid IgM, IgG, or IgG/IgM detection test. 2. Asymptomatic contact with a positive laboratory test for COVID-19.
Argentina	1. Confirmed by COVID-19 laboratory: any suspected cases with a detectable result for SARS-CoV-2 by molecular biology tests by RT-PCR, by molecular biology tests by loop-mediated isothermal amplification reaction (LAMP), or SARS-cov-2 antigens by non-molecular tests. Confirmatory diagnosis in suspected cases with mild or moderate symptoms, only during the first 7 days from the onset of symptoms. 2. Confirmed by clinical or epidemiologic criteria of COVID-19: in the last 14 days has been in close contact with a confirmed case OR Is part of a conglomerate of cases, with ≥1 case confirmed by laboratory, with no other defined diagnosis, and presenting ≥2 of the following symptoms: fever, cough, odynophagia, difficulty breathing, vomiting, diarrhea, headache, or myalgia. 3. Any person who, in the absence of any other identified cause, experiences sudden loss of taste or smell. 4. Any deceased person who does not have a defined etiologic diagnosis, has had a clinical picture compatible with COVID-19 (known by the certifying doctor or referred by third parties) regardless of previous health status and that has been close contact of a confirmed case or has been epidemiologically linked to a cluster of cases or to areas of sustained community transmission.
Spain	1. Person who meets clinical criteria for a suspected case and with a positive diagnostic test. 2. Asymptomatic person with positive diagnostic test with negative IgG or not performed.
Mexico	1. Person who meets the operational definition of suspected case and has a laboratory-confirmed diagnosis issued by Institute of Epidemiological Diagnosis and Reference. 2. Person who meets the operational definition of a suspected case, but who has been in contact with a laboratory confirmed case within the last 14 days from the date of onset of symptoms.
South Africa	A person with laboratory confirmation of SARS-CoV-2 infection (using an RT-PCR assay), irrespective of clinical signs and symptoms.
United Kingdom	Positive COVID-19 test result.
Iran	Laboratory confirmation with or without suspected or probable criteria.
Chile	1. Anyone who meets the definition of a suspected case in which the specific test for SARS-CoV-2 was positive (RT-PCR). 2. Any asymptomatic person identified through an active search strategy in which the specific test for SARS-CoV-2 was positive (RT-PCR).
Saudi Arabia	A person who meets the suspected case definition with laboratory confirmation of COVID-19 infection (PCR).

Country	Definition
Turkey	Cases in which SARS-CoV-2 is detected by molecular methods among the cases matching the possible case definition.
Indonesia	A person who has tested positive for the COVID-19 virus proven by the RT-PCR laboratory examination. Confirmation cases are divided into: confirmation case with symptoms (symptomatic), confirmation cases without symptoms (asymptomatic).
Israel	Positive COVID-19 test result.
Ukraine	A person with a laboratory-confirmed COVID-19 disease, regardless of clinical signs and symptoms.

*COVID-19, coronavirus disease; ECLIA, electrochemiluminescence immunoassay; ILI, influenza-like illness; MIS-C, multisystem inflammatory syndrome-children; NAAT, nucleic acid amplification technique; RT-PCR, reverse transcription PCR; SARS, severe acute respiratory syndrome; SARS-CoV-2, severe acute respiratory syndrome coronavirus 2.
†World Health Organization definition.
‡European Centre for Disease Prevention and Control definition.

Appendix Table 7. Full confirmed COVID-19 case definition criteria across 25 countries with the highest case counts, current as of January 1, 2021*

Country	Diagnostic testing						Clinical symptoms				Epidemiologic criteria			Other†
	PCR test	Antigen test	Antibody test	Positive test (nonspecified)	Radiographic imagery	Meet suspected case definition	Cough	Fever	SARI	Loss of taste or smell	Travel history	Hospitalized	Confirmed contact	Diagnostic, clinical, or EPI
WHO definition (reference)	X													
Argentina	X	X				X	X	X		X			X	X
Bangladesh‡	X													
Brazil	X	X	X		X				X	X			X	X
Chile	X					X				X		X		
Colombia	X	X												
France§	X	X												
Germany§	X	X												
India‡				X										
Indonesia	X													
Iran				X										
Iraq‡	X													
Israel				X										
Italy§	X	X												
Mexico				X		X				X			X	
Pakistan‡				X										
Peru	X	X				X					X	X	X	
Philippines‡	X	X												
Russia	X	X	X											
Saudi Arabia	X					X					X	X	X	
South Africa	X													
Spain	X	X				X				X				
Turkey	X					X				X	X	X	X	
Ukraine				X										
United Kingdom				X										
USA	X													
Totals	18	10	2	7	1	7	1	1	1	6	3	4	6	2
% of countries¶	72%	40%	8%	28%	4%	28%	4%	4%	4%	24%	12%	16%	24%	8%

*X indicates the criterion was sufficient for, or a potential component of, the confirmed case definition requirement(s). Full confirmed case definitions can be found in Appendix Table 6.
†Refer to probable case definition for applicable country (Appendix Table 6).
‡World Health Organization definition (confirmed case definition did not change between March 2020 and August 2020 update).
§European Centre for Disease Prevention and Control definition.
¶Denominator is 25 countries with confirmed case definitions.

Appendix Table 8. COVID-19 testing policies for asymptomatic persons in 25 countries with highest case counts as of January 1, 2021*

Countries	Asymptomatic testing	EPI Criteria: Confirmed contact	EPI Criteria: Healthcare employee
WHO	Yes with EPI Criteria	Yes	
Iraq	Yes with EPI Criteria	Yes	
Bangladesh	No		
Philippines	Yes		
India	Yes with EPI Criteria	Yes	Yes
Pakistan	Yes		
United States	Yes		
Italy	Yes		
Germany	Yes with EPI Criteria	Yes	Yes
France	Yes		
Brazil	Yes with EPI Criteria	Yes	Yes
Russia	Yes		
Colombia	No		
Peru	No		
Argentina	No		
Spain	Yes with EPI Criteria	Yes	Yes
Mexico	No		
South Africa	Yes with EPI Criteria	Yes	
United Kingdom	No		Yes
Iran	Yes with EPI Criteria	Yes	
Chile	Yes		
Saudi Arabia	No		Yes
Turkey	Yes with EPI Criteria	Yes	Yes
Indonesia	No		
Israel	Yes		
Ukraine	No		

*EPI, epidemiologic; WHO, World Health Organization.

References

1. World Health Organization. Public health surveillance for COVID-19: interim guidance, 7 August 2020 [cited 2020 Dec 13]. https://apps.who.int/iris/handle/10665/333752

2. Argentina Ministry of Health. Case definition [in Spanish]. 2020 [cited 2020 Jan 4]. https://www.argentina.gob.ar/salud/coronavirus-COVID-19/definicion-de-caso

3. Argentina Ministry of Health. Consensus on the use of diagnostic tests for SARS-CoV-2 [in Spanish] [cited 2020 Dec 4]. https://bancos.salud.gob.ar/sites/default/files/2020-09/covid-19-consenso-sobre-uso-de-pruebas-diagnosticas-para-sars-cov-2.pdf

4. Government of the People's Republic of Bangladesh. National guidelines on clinical management of COVID-19. 2020 [cited 2020 Dec 4]. https://covidlawlab.org/wp-content/uploads/2021/01/Bangladesh_2020.11.05_Guideline_National-Guidelines-on-Clinical-Management-of-COVID-19_EN.pdf

5. Ministry of Health. Brazil. Epidemiological surveillance guide public health emergency of national importance for coronavirus disease 2019 [in Portuguese]. 2020 Aug 5 [cited 2020 Dec 3]. https://portalarquivos.saude.gov.br/images/af_gvs_coronavirus_6ago20_ajustes-finais-2.pdf

6. Narbona P. Update on the definition of a suspected, confirmed and probable case for epidemiological surveillance in the face of a COVID-19 pandemic [in Spanish]. 2020 Oct 1 [cited 2021 Jan 4]. https://www.minsal.cl/wp-content/uploads/2020/10/201006-Definici%C3%B3n-de-caso-sospechoso.pdf

7. Government of Chile. Action plan for coronavirus [in Spanish]. 2020 [cited 2020 Nov 30]. https://www.gob.cl/coronavirus

8. Ministry of Health and Social Protection, Government of Colombia. Optimization of testing, tracking and isolation program for the monitoring and follow-up of COVID-19 cases and contacts in Colombia [in Spanish]. 2020 Oct 19 [cited 2020 Nov 30]. https://www.minsalud.gov.co/Normatividad_Nuevo/Decreto%201374%20de%202020.pdf

9. Ministry of Health and Social Protection, Government of Colombia. Testing, tracking and sustainable selective insulation [in Spanish]. 2020 [cited 2020 Dec 4]. https://www.minsalud.gov.co/sites/rid/Lists/BibliotecaDigital/RIDE/VS/ED/VSP/abece-prass.pdf

10. European Centre for Disease Prevention and Control. Case definition for coronavirus disease 2019 (COVID-19), as of 29 May 2020 [cited 2020 Sep 16]. https://www.ecdc.europa.eu/en/covid-19/surveillance/case-definition

11. Ministry of Solidarities and Health, Government of France. RT-PCR, antigenic and salivary tests [in French]. 2020 [cited 2020 Dec 3]. https://solidarites-sante.gouv.fr/soins-et-maladies/maladies/maladies-infectieuses/coronavirus/tout-savoir-sur-la-covid-19/article/les-tests-rt-pcr-antigeniques-et-salivaires

12. Federal Ministry of Health. Germany. The national test strategy. 2020 [cited 2021 Jan 5]. https://www.zusammengegencorona.de/en/die-nationale-teststrategie/

13. Government of India. Clinical management protocol for COVID-19. 2020 [cited 2020 Oct 25]. https://www.mohfw.gov.in/pdf/UpdatedClinicalManagementProtocolforCOVID19dated03072020.pdf

14. Indian Council of Medical Research, Government of India. Advisory on strategy for COVID-19 testing in India. 2020 Sep 4 [cited 2020 Nov 25]. https://www.mohfw.gov.in/pdf/AdvisoryonstrategyforCOVID19TestinginIndia.pdf

15. Republic of Indonesia. Prevention and control guidelines coronavirus disease 2019 (COVID-19) [in Indonesian]. 2020 Jul 13 [cited 2020 Nov 27]. https://covid19.go.id/p/protokol/pedoman-pencegahan-dan-pengendalian-coronavirus-disease-covid-19-revisi-ke-5

16. Government of Indonesia. Q&A [in Indonesian]. 2020 [cited 2020 Dec 3]. https://covid19.go.id/tanya-jawab?page=2

17. Israel Ministry of Health. Testing for COVID-19. 2020 [cited 2020 Nov 30]. https://www.gov.il/en/Departments/General/corona-tests

18. Minister of Health, Government of Italy. Operational guidelines for carrying out rapid antigen tests by general practitioners and pediatricians of free choice [in Italian]. 2020 [cited 2021 Jan 6]. https://www.trovanorme.salute.gov.it/norme/renderNormsanPdf?anno=2020&codLeg=76980&parte=1%20&serie=null

19. Secretary of Health, Government of Mexico. Standardized guidelines for the epidemiological and laboratory surveillance of viral respiratory disease [in Spanish]. 2020 [cited 2020 Jan 4]. https://coronavirus.gob.mx/wp-content/uploads/2020/09/Lineamiento_VE_y_Lab_Enf_Viral_Ago-2020.pdf

20. Government of Mexico. Update of the operational definition of suspicious case of viral respiratory disease [in Spanish]. 2020 Aug 24 [cited 2020 Sep 16]. https://www.gob.mx/cms/uploads/attachment/file/573732/Comunicado_Oficial_DOC_sospechoso_ERV_240820.pdf

21. Secretary of Health, Government of Mexico. Emerging statement on the use of tests for the detection of SARS-CoV-2 antigen in Mexico [in Spanish]. 2020 Nov 11 [cited 2020 Dec 3]. https://coronavirus.gob.mx/wp-content/uploads/2020/11/Prueba_antigenica_COVID_11Nov2020.pdf

22. National Institute of Health. Pakistan. Case definition for COVID-19. 2020 [cited 2020 Nov 27]. https://www.nih.org.pk/wp-content/uploads/2020/03/Case-Definition-for-COVID-19.pdf

23. Ministry of National Health Services. Pakistan. National testing guidelines: real-time polymerase chain reaction (RT-PCR) diagnostic test. 2020 [cited 2021 Dec 7]. https://storage.covid.gov.pk/new_guidelines/02July2020_20200701_National_Testing_Guidelines_for_(RT-PCR)_Diagnostic_Test_0103.pdf

24. Ministry of National Health Services. Pakistan. Testing Strategy Incorporating COVID-19 Antigen Detection Rapid Diagnostic Tests (Ag-RDT). 2020 [cited 2020 Jan 5]. https://storage.covid.gov.pk/new_guidelines/02December2020_20201202_TESTING_STRATEGY_INCORPORATING_COVID-19_ANTIGEN_DETECTION_RAPID_DIAGNOSTIC_TESTS_(Ag-RDT)_5302.pdf

25. Government of Peru. Epidemiological alert to the risk of intensification of community transmission of COVID-19 in the post-quarantine period, in Peru [in Spanish]. 2020 [cited 2020 Jan 4]. https://www.dge.gob.pe/portal/docs/alertas/2020/AE019.pdf

26. Government of Peru. Epidemiological alert for the application of diagnostic tests in cases of COVID-19 in Peru [in Spanish]. 2020 [cited 2020 Sep 30]. https://www.dge.gob.pe/portalnuevo/publicaciones/alertas-epidemiologicas/

27. Republic of the Philippines Department of Health. Further amendment to administrative order no. 2020–0013 dated 09 April 2020 entitled, "Revised administrative order no. 2020-0012, 'guidelines for the implementation for the inclusion of the coronavirus disease 2019 (COVID-19) in the list of notifiable diseases for mandatory reporting to the Department of Health' dated March 17, 2020." 2020 [cited 2020 Jan 4]. https://doh.gov.ph/sites/default/files/health-update/ao2020-0013-B.pdf

28. Republic of the Philippines Department of Health. Amendment to department memorandum no. 2020–0258 entitled updated interim guidelines on expanded testing for COVID-19. 2020 Jul 6 [cited 2020 Dec 07]. https://www.doh.gov.ph/sites/default/files/health-update/dm2020-0258-A.pdf

29. Russian Federation. Temporary methodological recommendations prevention, diagnosis and treatment of new coronavirus infection (COVID-19) [in Russian]. 2020 [cited 2020 Dec 07]. https://xn--80aesfpebagmfblc0a.xn--p1ai/ai/doc/699/attach/mr_COVID-19_v9_.pdf

30. Russian Federation. Frequently asked questions. 2020 [cited 2020 Nov 30]. https://xn--80aesfpebagmfblc0a.xn--p1ai/faq/?tags=39

31. Kingdom of Saudi Arabia. COVID-19 coronavirus disease guidelines. 2020 [cited 2020 Dec 14]. https://covid19.cdc.gov.sa/wp-content/uploads/2020/10/EN_COVID_19_Coronavirus_Disease_Guidelines_v2.0.pdf

32. Republic of South Africa. Coronavirus disease 2019 (COVID-19) caused by a novel coronavirus (SARS-CoV-2). 2020 [cited 2020 Nov 25]. https://www.nicd.ac.za/wp-content/uploads/2020/09/Guidelines-for-case-finding-diagnosis-and-public-health-response-in-South-Africa_18Aug2020.pdf

33. Republic of South Africa Department of Health. Statement by President Cyril Ramaphosa on progress in the national effort to contain the COVID-19 pandemic. 2020 Sep 16 [cited 2020 Dec 07]. https://sacoronavirus.co.za/2020/09/16/statement-by-president-cyril-ramaphosa-on-progress-in-the-national-effort-to-contain-the-covid-19-pandemic-3/

34. Government of Spain. Early detection, surveillance and control of COVID-19 [in Spanish]. 2020 [cited 2020 Jan 4]. https://www.mscbs.gob.es/profesionales/saludPublica/ccayes/alertasActual/nCov/documentos/COVID19_Estrategia_vigilancia_y_control_e_indicadores.pdf

35. Republic of Turkey Ministry of Health. COVID-19 (SARS-CoV-2 infection) general information, epidemiology, and diagnosis [in Turkish]. 2020 [cited 2020 Dec 8]. https://covid19.saglik.gov.tr/Eklenti/39551/0/covid-19rehberigenelbilgilerepidemiyolojivetanipdf.pdf

36. Ministry of Health of Ukraine. Organization of medical care for patients with coronavirus disease (COVID-19) [in Ukrainian]. 2020 [cited 2021 Jan 04]. https://moz.gov.ua/article/ministry-mandates/nakaz-moz-ukraini-vid-28032020--722-organizacija-nadannja-medichnoi-dopomogi-hvorim-na-koronavirusnu-hvorobu-covid-19

37. Ministry of Health of Ukraine. COVID-19 pandemic in Ukraine [cited 2020 Dec 8]. https://covid19.gov.ua/en

38. UK Healthy Security Agency. COVID-19: investigation and initial clinical management of possible cases. 2020 [cited 2020 Nov 25]. https://www.gov.uk/government/publications/wuhan-novel-coronavirus-initial-investigation-of-possible-cases/investigation-and-initial-clinical-management-of-possible-cases-of-wuhan-novel-coronavirus-wn-cov-infection

39. UK National Health Service. Get tested for coronavirus (COVID-19) [cited 2020 Nov 30]. https://www.gov.uk/getting-tested-for-coronavirus

40. Centers for Disease Control and Prevention. Coronavirus Disease. 2019 (COVID-19) 2020 interim case definition, approved August 5, 2020. 2020 [cited 2020 Oct 13]. https://ndc.services.cdc.gov/case-definitions/coronavirus-disease-2019-2020-08-05/

41. Centers for Disease Control and Prevention. Overview of testing for SARS-CoV-2 (COVID-19). 2020 [cited 2020 Dec 07]. https://www.cdc.gov/coronavirus/2019-ncov/hcp/testing-overview.html.
